# Prevalence and outcomes of hypocalcemia on ED arrival in traumatic patients before blood transfusions: a systematic review and meta-analysis

**DOI:** 10.1186/s13049-025-01361-y

**Published:** 2025-03-17

**Authors:** Wuttipong Srichuachom, Sarunsorn Krintratun, Boriboon Chenthanakij, Wachira Wongtanasarasin

**Affiliations:** Department of Emergency Medicine, Faculty of Medicine, Chiang Mai University, 110 Intavarorot Street, Sriphum, Chiang Mai, 50200 Thailand

**Keywords:** Hypocalcemia, Hemorrhagic shock, Trauma, Mortality, Systematic review

## Abstract

**Background:**

Hypocalcemia represents a critical physiological disturbance in trauma-related hemorrhagic shock patients. Nonetheless, the prevalence and effects of hypocalcemia remain uncertain. This study aims to explore the prevalence of hypocalcemia before blood transfusions and its related mortality in adult patients with major traumatic injuries.

**Method:**

We conducted a systematic search through databases, including PubMed, EMBASE, Scopus, and Web of Science, from their inception until June 30, 2024. Patients with major traumatic injuries whose serum calcium was measured upon arrival at the emergency department (ED) were included. We excluded those with prior blood transfusions, pre-clinical studies, review articles, and studies without a control group. Meta-analysis using a random-effect model was performed. Heterogeneity was evaluated using Cochrane Q and I² statistics. The study’s risk of bias was assessed using the Joanna Briggs Institute’s critical appraisal checklist. Publication bias was assessed using Egger’s test and contour funnel plot visualization.

**Results:**

Of the total, 1,984 abstracts were screened, leading to 15 studies in this review and meta-analysis. The overall prevalence of hypocalcemia upon ED arrival was 56% (95% CI 37%-74%), with high heterogeneity (*I*^2^ 99.8%) observed. Hypocalcemia patients also had higher mortality rates than normocalcemia patients (OR 2.44, 95% CI 1.76–3.40).

**Conclusion:**

Hypocalcemia on ED arrival is common among adult trauma patients, who also exhibit a notably high mortality rate within this demographic. We recommend the monitoring of ionized calcium levels in traumatic adult patients. Furthermore, systematically designed studies examining the optimal thresholds, treatment protocols, and outcomes should be prioritized as the focal point of research.

**Trial registration:**

CRD42024549054 (http://www.crd.york.ac.uk/PROSPERO).

**Supplementary Information:**

The online version contains supplementary material available at 10.1186/s13049-025-01361-y.

## Introduction

Injury is a significant contributor to death and disability worldwide [1]. It imposes a substantial burden through economic costs and long-term health disabilities [1, 2]. While traumatic brain injury is the most significant global contributor to trauma mortality overall, hemorrhagic shock remains a leading cause of preventable early deaths in major trauma patients, contributing to nearly 1.5 million deaths globally [3, 4]. Deaths from hemorrhage typically occur in the early phase of injury, with a median time of around two hours post-injury [2, 3]. However, early hypotension is associated with delayed mortality, such as from organ failure or infection [5] —Currently, treatments for hemorrhagic shock focus on bleeding control and achieving definitive hemostasis [3, 6].

Ionized calcium is a form of calcium in the human body that can be measured using portable devices [7]. Calcium is crucial in regulating vasomotor tone, platelet function, the coagulation cascade, and cardiac contractility, all vital in trauma settings [7]. In trauma patients, hypocalcemia has been studied and found to be correlated with poor outcomes, such as higher mortality and an increased need for massive transfusion [8–12]. Despite these associations, there is no definitive conclusion on the direct impact of hypocalcemia on trauma outcomes [7, 13].

The occurrence of hypocalcemia in major trauma patients varies across studies, and its impact on patient outcomes is still unclear [14–17]. Therefore, we conducted a systematic review and meta-analysis to establish the incidence of initial hypocalcemia in adult traumatic patients without prior blood product administration. Additionally, we also aimed to investigate the relationship between hypocalcemia and mortality in these patients.

## Methods

This systematic review was prepared following the Preferred Reporting Items for Systematic Reviews and Meta-Analyses statements [18]. We prospectively registered the study protocol with the PROSPERO website before collecting data (Registration ID: CRD42024549054, http://www.crd.york.ac.uk/PROSPERO).

### Search strategy and study selection

We systematically searched four databases, PubMed, EMBASE, Scopus, and Web of Science, from their inception until June 30, 2024. Our search strategy did not impose any language restrictions. We employed a combination of the Medical Subject Heading (MeSH) terms, along with different spellings and endings, to identify relevant articles on “hypocalcemia,” “injuries,” “wound,” “incidence,” “prevalence,” “trauma,” and “emergency room.” Detailed search terms were described in Supplementary files. We also searched websites, organizations, relevant reviews, and references to identify additional eligible studies. We also searched for citations from relevant articles. The search results from these databases were extracted. Duplicate studies were removed, and the remaining studies were added to the Rayyan QCRI website. Rayyan website (https://rayyan.qcri.org) was used to streamline the systematic review process by assisting with the screening of titles and abstracts and anonymously selecting eligible and inclusion studies [19].

### Inclusion criteria and outcome of interest

The inclusion criteria were as follows:

1. Any study included traumatic adult patients (Age > 15) with no prior blood transfusion before taking iCa level who presented to the emergency department (ED).
2. Reporting of prevalence or incidence of hypocalcemia on arrival at the ED (i.e., did not receive any blood product).
3. Reporting of outcome or endpoint on mortality of hypocalcemia and normocalcemia groups.

Hypocalcemia was defined by low calcium levels in the blood serum measured by either ionized calcium (iCa) or total calcium (Ca) levels. We excluded pre-clinical studies, review articles, and studies without a control group (i.e., case reports, case series, etc.). We (W.S. and W.W.) independently screened study titles and abstracts to identify potentially eligible studies. Full-text articles of the retrieved studies were extracted and independently assessed by two authors against the pre-specified criteria. Any discrepancies were discussed with another author (S.K.) and resolved through consensus. The primary outcome was the prevalence of hypocalcemia on arrival at the ED. The secondary outcome included hospital mortality between hypocalcemia and normocalcemia groups.

### Data extraction and assessment of the study risk of bias

Data was extracted from the included articles following a pre-specified data extraction form, including the first author, publication year, country, study setting, study duration, cohort size, numbers of hypocalcemia, key inclusion and exclusion criteria, injury severity score (ISS), and mortality. All extracted data were entered into a data spreadsheet (Microsoft Excel). Two authors (W.S. and S.K.) independently assessed the risk of bias in the included studies, and any disagreements were resolved by the third author (W.W.). The study risk of bias was assessed using the Joanna Briggs Institute’s (JBI) critical appraisal checklist [20, 21].

### Statistical analysis

We collected the relevant information in the prepared data spreadsheet. The extracted database was exported to Stata MP 16 statistical software (StataCorp LLC, College Station, TX) for statistical analysis. We estimated the prevalence of hypocalcemia and the corresponding 95% confidence interval (CI). The random-effects model was used to adjust for predicted significant heterogeneity among studies. Subgroup analyses were performed based on the study risk of bias (“low risk” as having a JBI checklist of 8–9, “some concern” as having a JBI checklist of 5–7, and “high risk” as having a JBI checklist of 0–4. We calculated the pooled odds ratio (OR) and corresponding 95% CI between groups using the random-effect REML model for the secondary outcome. We also performed sensitivity analyses to assess the direction of the relationship between the severity of hypocalcemia and mortality rates in these patients. The publication bias was assessed using Egger’s test and visualization of the contoured Funnel plot. Heterogeneity among studies was evaluated using Cochrane Q and the I^2^ statistics. All tests were two-sided, with a p-value of less than 0.05, which was considered statistically significant.

## Results

### Study selection and characteristics

**Fig. 1:**
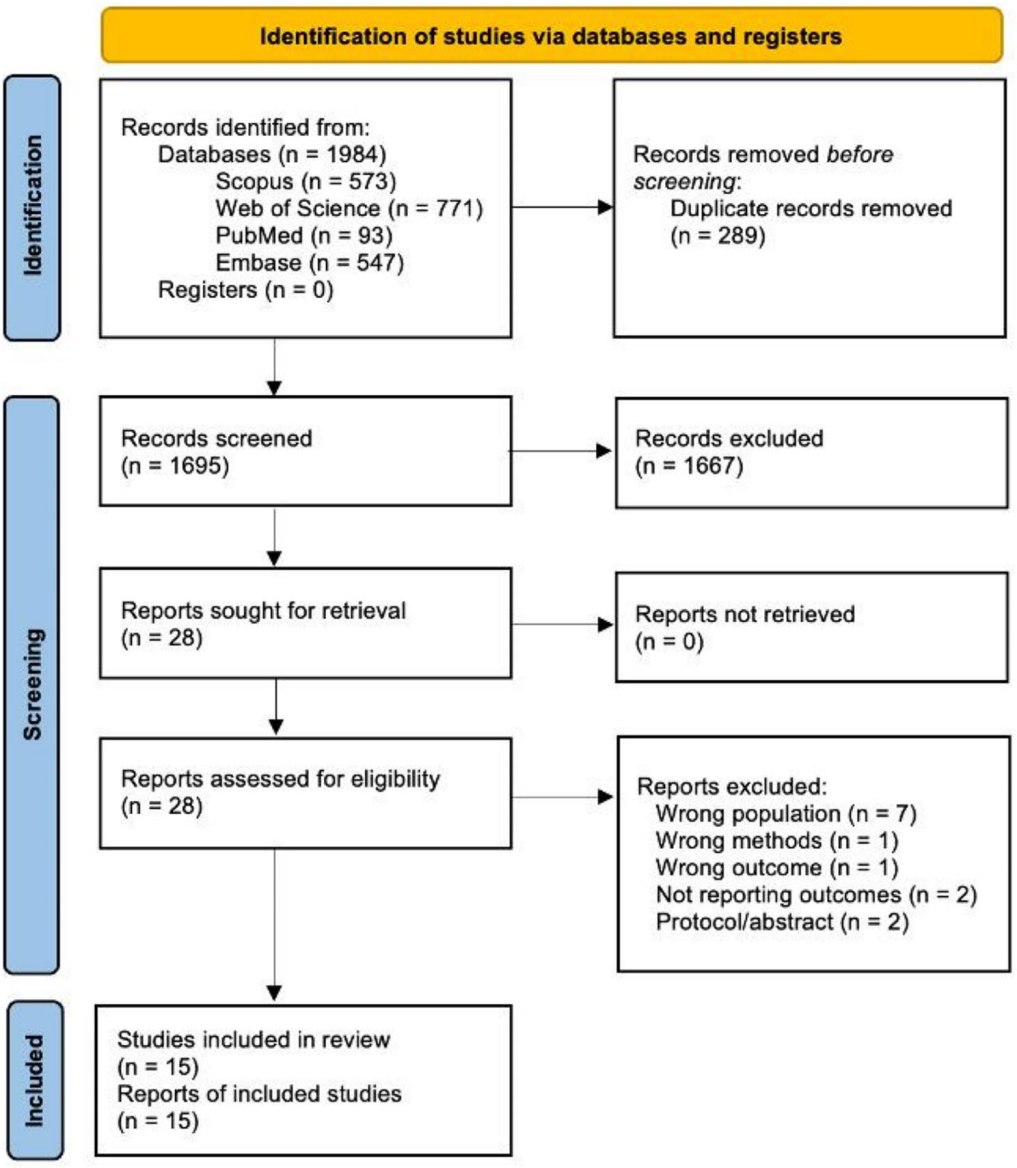
PRISMA diagram of searching, screening, and inclusion

A total of 1,984 abstracts were preliminary screened through a search of four databases (Fig. 1). Of these, 28 full-text studies were examined, and thirteen were excluded. This resulted in fifteen studies included in this review and meta-analysis [8–12, 14–17, 22–27]. All observational cohort studies (Table 1) were undertaken at a trauma center. Studies varied in sample size (*n =* 60–30,183), inclusion and exclusion criteria (Table S1), definition of hypocalcemia, and prevalence of blunt injury (73 -100%).

**Table 1:** Details of included studies and summary of patient characteristics

No	Study Country	Study type	Study duration	Setting	Cohort size (*n*)	Patients with hCa (*n*)	Patients with shCa (*n*)	Definitions of hCa and shCa	Age– years, mean ± SD	Male (%)	Blunt injury (%)	ISS, median (IQR)		Mortality (%)	
												hCa	Normal Ca	hCa	Normal Ca
1	Vivien, 2005 France	Prospective observational cohort	2002	Level 1 academic trauma center	212	156	21	hCa: iCa < 1.15 mmol/L shCa: iCa < 0.9 mmol/L	37 ± 16	74	98	34 (29–36)^a^ 59 (41–75)^b^	16 (12–24)	19^a^, 71^b^	5
2	Cherry, 2006 USA	Retrospective observational cohort	2000–2002	Level 1 trauma center	396	91	N/A	iCa < 1 mmol/L	37 ± 5	N/A	N/A	25 (13–34)	22 (13–34)	26	16
3	Choi, 2008 Korea	Ambispective observational cohort	2005	University hospital	255	248	53	hCa: iCa < 1.15 mmol/L shCa: iCa < 0.88 mmol/L	47 ± 16	77	95	N/A	N/A	11^a^, 30^b^	14
4	Magnotti, 2011 USA	Prospective observational cohort	2008	Level 1 trauma center	591	332	N/A	iCa < 1 mmol/L	38^c^	78	78	23	18	16	9
5	Webster, 2016 UK	Retrospective observational cohort	2013–2014	Trauma audit research network	55	30	N/A	iCa < 1.1 mmol/L	33^c^	65	82	N/A	N/A	N/A	N/A
6	Vasudeva, 2019 Australia	Retrospective observational cohort	2014–2018	Level 1 trauma center	226	113	6	hCa: iCa < 1.1 mmol/L shCa: iCa < 0.8 mmol/L	44 ± 21	66	86	38 (24–43)	26 (17–34)	26	15
7	Byerly, 2020 USA	Retrospective observational cohort	2004	Level 1 trauma center	7,341	N/A	716	shCa: iCa < 0.9 mmol/L	39 (26–55)^c^	81	51	25 (14–34)^b^	14 (9–22)	38^b^	10
8	Helshoot, 2023 Multi-center	Retrospective observational cohort	2015–2019	Trauma registry DGU	30,183	3,982	240	hCa: iCa < 1.1 mmol/L shCa: iCa < 0.9 mmol/L	54 (35–70)^c^	71	93	22 (16–29)	20 (14–29)	21^a^, 36^b^	14
9	Scahid Jr, 2023 USA	Prospective observational cohort	N/A	Urban level 1 trauma center	68	16	N/A	iCa < 1 mmol/L	37 ± 18	81	81	22 (15–34)	13 (8–24)	12	8
10	Badarni, 2023 Israel	Retrospective observational cohort	2014–2020	Level 1 trauma center	201	147	13	hCa: iCa < 1.16 mmol/L shCa: iCa < 1 mmol/L	53 (28–69)^c^	82	95	29 (25–30)	26 (25–29)	20	19
11	Maekkodathil, 2023 Qatar	Retrospective observational cohort	2016–2021	Level 1 trauma center	922	757	N/A	Ca < 2.2 mmol/L	32 ± 15	94	N/A	23 ± 11^d^	28 ± 10^d^	24	12
12	Vettorello, 2023 Italy	Retrospective observational cohort	2015–2021	Level 1 trauma center	798	129	N/A	iCa < 1.11 mmol/L	47 ± 20	74	100	38 (28–50)	26 (20–33)	13	3
13	Ahmed, 2024 Egypt	Prospective observational cohort	2022	Level 1 trauma center	60	30	N/A	iCa < 1.11 mmol/L	46 ± 18	87	100	N/A	N/A	73	30
14	Ciaraglia, 2024 USA	Retrospective observational cohort	2016–2019	Trauma registry	1,981	869	N/A	iCa < 1 mmol/L	40 (27–58)^c^	73	73	N/A	N/A	16	11
15	Liaud-Laval, 2024 France	Retrospective observational cohort	2015–2021	Level 1 trauma center	137	134	23	hCa: iCa < 1.2 mmol/L shCa: iCa < 0.9 mmol/L	39 (23–55)^c^	76	75	26 (17–34)^a^ 34 (27–40)^b^	26 (19–34)	39^b^	N/A

All numbers in the table have been rounded

^a^ for patients with mild hypocalcemia, ^b^ for patients with severe hypocalcemia, ^c^ reported as median (IQR, if available), ^d^ reported as mean ± SD

Abbreviations: hCa, hypocalcemia; iCa, ionized calcium; N/A, not applicable; shCa, severe hypocalcemia

### Risk of bias in studies

Eight studies were classified as having a low risk of bias, while the remaining had some concerns (Table S2). Bias was mainly due to the data needed to be collected with sufficient coverage of the identified sample [8, 9, 12, 14–16, 22, 23, 25]. Moreover, the selection of patients included in the studies also risked assessing hypocalcemia since they targeted only traumatic brain injury patients [12, 22, 25].

### Primary and secondary outcome

**Fig. 2:**
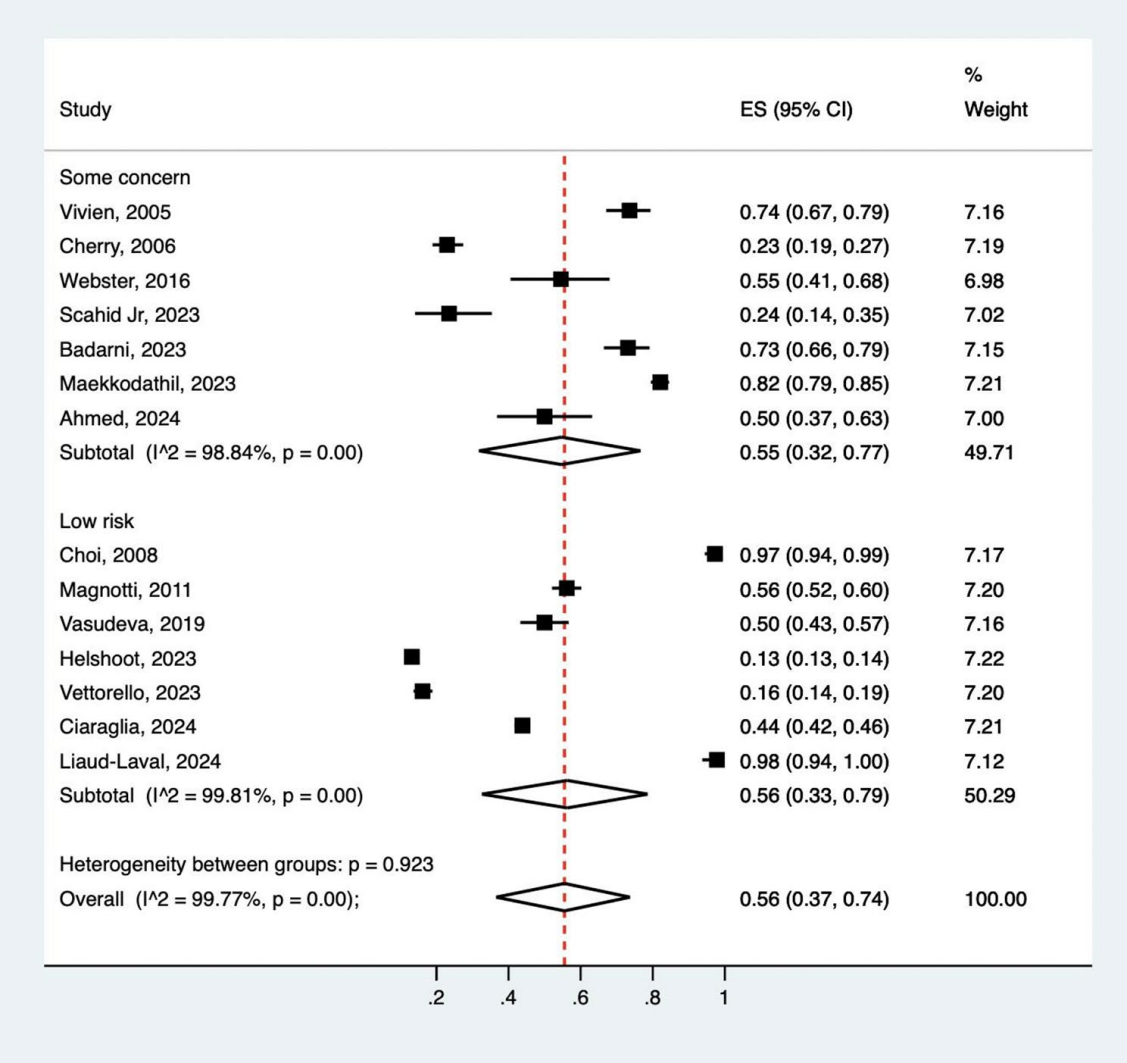
Forest plot of incidence of hypocalcemia in traumatic adult patients, which subgroups as some concern and low risk of bias

Hypocalcemia was defined differently among the included studies (Table 1). The definition varied from < 0.9 mmol to < 1.16 mmol/L. The overall prevalence of hypocalcemia on arrival at the ED was 56% (95% CI 37%-74%, Fig. 2). Notably, this synthesis was largely heterogeneous (*I*^2^ 99.8%), with no significant difference observed among low-risk and some concern studies (*p* = 0.92). In addition, the prevalence of severe hypocalcemia on arrival at the ED was 8% (95% CI 3%-16%, Figure S1).

**Fig. 3:**
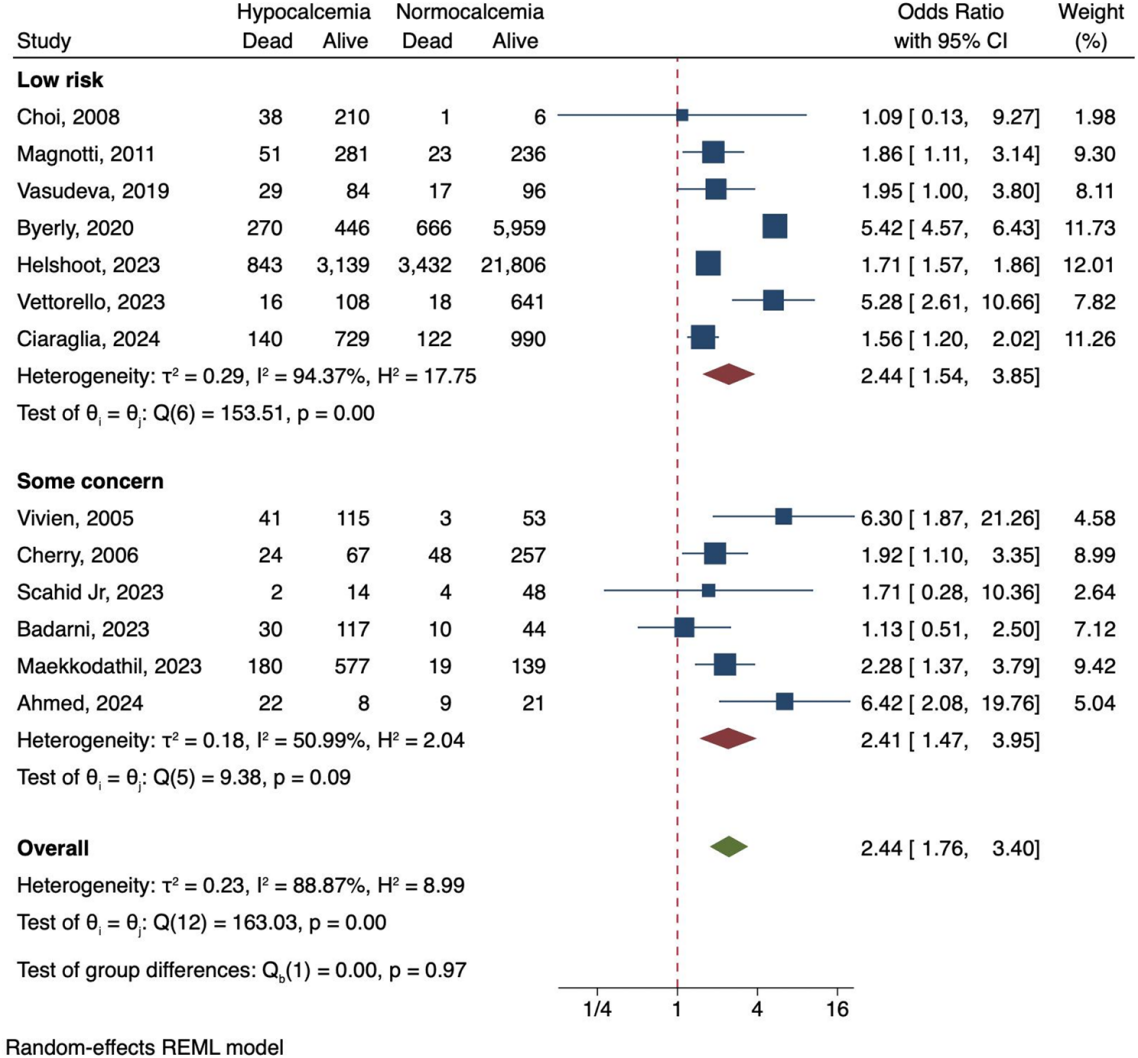
Forest plot of hypocalcemia and mortality rate in traumatic adult patients using random-effects model, which subgroups as some concern and low risk of bias

Among thirteen studies that reported mortality, patients with hypocalcemia on ED arrival had higher mortality rates than normocalcemia patients (OR 2.44, 95% CI 1.76–3.40, Fig. 3). No difference was observed among low-risk and some concern studies (*p* = 0.97). High heterogeneity was observed in each group (based on the risk of bias, *I*^2^ 94.4% in low-risk studies and 51.0% in some concern studies). Additionally, we found a similar direction, considering three studies reported mortality in patients with severe hypocalcemia. They had higher mortality compared with patients with normocalcemia (OR 7.46, 95% CI 1.35–41.26, Figure S2).

### Reporting bias

**Fig. 4:**
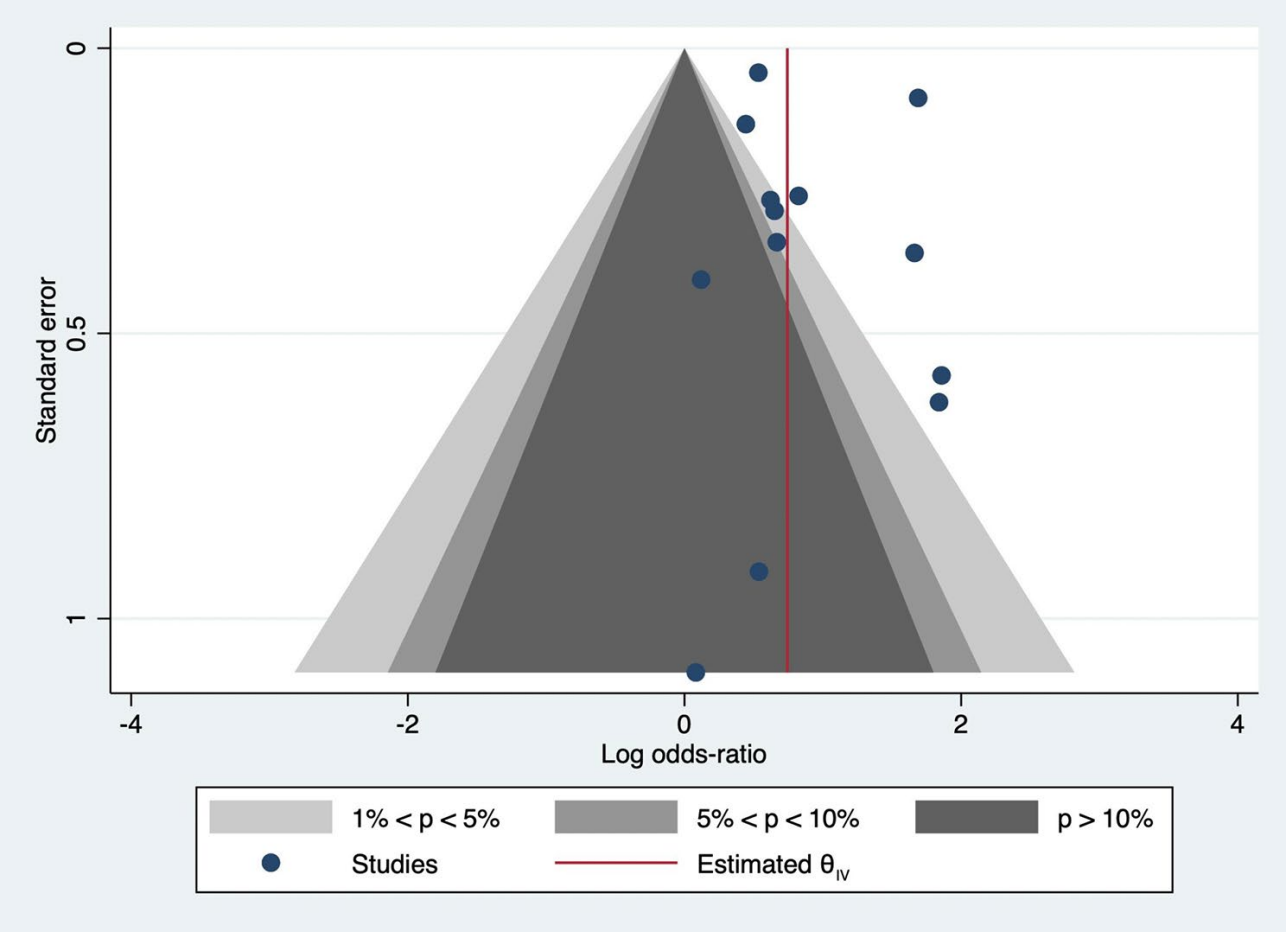
Contoured funnel plot shows no asymmetry

The contoured funnel plot shows no asymmetry (Fig. 4). The distribution of studies within and outside the contour lines was similar, suggesting no potential publication bias. Egger’s test confirmed this observation with a p-value of 0.86.

## Discussion

This systematic review and meta-analysis represents an updated comprehensive overview of hypocalcemia in adult major trauma patients. By integrating retrospective and prospective observational studies, we expanded upon previous research [14] to encompass a wider range of patient subgroups [22, 25, 26]. Our findings revealed that hypocalcemia is a common condition affecting approximately half of adult major trauma patients. This finding aligns with prior studies demonstrating an association between major trauma and hypocalcemia, even after blood transfusion [11, 28]. Several mechanisms may contribute to the development of hypocalcemia in trauma patients, including citrate chelation [7, 29], acidosis [9], and parathyroid hormone dysfunction [30–33]. Based on these findings, we recommend monitoring ionized calcium levels for all adult major trauma patients upon hospital admission. Furthermore, the mechanisms underlying hypocalcemia in trauma patients, beyond those associated with transfusion, warrant further investigation to address the existing gaps in knowledge.

Our study further highlights a significant association between hypocalcemia (also severe hypocalcemia) and increased mortality rates, consistent with previous research [3, 13, 14, 28]. However, we acknowledge that the relationship between hypocalcemia and mortality is not as straightforward as initially concluded. For instance, the Helsloot study [14], the largest included study, suggests a bimodal relationship between hypocalcemia and mortality. This indicates that while low calcium levels are associated with worse outcomes, patients with hypercalcemia also resulted in increased mortality. This phenomenon may stem from various factors, such as muscle breakdown or skeletal fractures, though the exact pathophysiological mechanisms remain poorly understood. Several hypotheses attempt to explain this relationship, including the theory that cytosolic and mitochondrial calcium overload disrupt critical intracellular signaling pathways, ultimately triggering cell death mechanisms [34, 35]. This bimodal relationship highlights the potential harm of unrecognized hypercalcemia, particularly in the context of excessive calcium supplementation. Further studies are needed to explore this potential bimodal relationship and its implications for clinical practice.

Hypocalcemia could indeed reflect injury severity rather than being a direct cause of mortality. Several hypotheses can explain the pathophysiological link. For example, hypocalcemia might exacerbate trauma-induced coagulopathy through its role in the coagulation cascade [14], leading to worsened hemostasis. Additionally, calcium is critical for cardiac contractility and vascular tone [36–38], and hypocalcemia-induced hypotension and impaired myocardial function could further compromise patients with major trauma [36–41]. Moreover, citrate chelation from transfusions and acidosis may contribute to reduced ionized calcium levels [42], compounding systemic effects. To address these gaps, future research should aim to dissect whether hypocalcemia serves as a marker of injury severity or actively contributes to adverse outcomes. Studies focusing on the temporal relationship between hypocalcemia onset and clinical deterioration, alongside controlled trials investigating calcium supplementation strategies, are essential to clarify its causal role and therapeutic implications in trauma patients.

While our study demonstrates no evidence of publication bias, it is important to acknowledge its limitations. First, there is significant heterogeneity in the diagnostic criteria, study settings, patient populations, and timing of calcium measurement across the included studies. For instance, while our inclusion criteria specified that calcium levels should be measured on admission before transfusion, variations in the timing and methodology among the included studies remain a limitation, as this can influence the generalizability of our findings. Second, many of the included studies are single-center with small cohorts, potentially impacting the statistical power and external validity of our results. Furthermore, the risk of bias assessment, although conducted rigorously, may underestimate the actual bias due to inconsistencies in reporting across studies. We also note that the included studies are highly heterogeneous in their methodologies and timing for measuring calcium levels. Importantly, most studies did not account for pH adjustments, which is a critical limitation. A low pH, often seen in critically ill trauma patients, can influence ionized calcium levels. Without pH adjustment, calcium levels might primarily serve as a marker of critical illness rather than representing a physiological or therapeutic target. This limitation underscores the need for future research to standardize calcium measurement methods, including pH adjustments, and to better delineate the role of calcium levels as a potential therapeutic target in trauma care.

We agree that a meta-analysis requires a well-defined patient population. The heterogeneity identified in our included studies underscores the need for future research to focus on standardizing diagnostic criteria and reporting methods. Despite these limitations, our findings emphasize the need for monitoring and managing ionized calcium levels in major trauma patients and highlight the critical need for large-scale, multicenter, randomized controlled trials to establish standardized thresholds and treatment protocols for hypocalcemia in trauma care.

## Conclusion

Hypocalcemia at ED arrival frequently occurs in adult trauma patients before blood transfusions and is associated with increased mortality. While definitive diagnostic criteria are absent, we suggest monitoring ionized calcium levels in this population. In the future, well-structured studies should focus on optimal thresholds, treatment protocols, and endpoints for calcium supplementation.

## Electronic supplementary material

Below is the link to the electronic supplementary material.

Supplementary Material 1

Supplementary Material 2

## Data Availability

No datasets were generated or analysed during the current study.

## References

[1] Kauvar DS, Wade CE. The epidemiology and modern management of traumatic hemorrhage: US and international perspectives. Crit Care. 2005;9(Suppl 5):S1–9. 10.1186/cc3779 PMC3226117 16221313

[2] Kauvar DS, Lefering R, Wade CE. Impact of hemorrhage on trauma outcome: an overview of epidemiology, clinical presentations, and therapeutic considerations. Infection and Critical Care: Journal of Trauma - Injury; 2006. 10.1097/01.ta.0000199961.02677.19 16763478

[3] Latif RK, Clifford SP, Baker JA, Lenhardt R, Haq MZ, Huang J et al. Traumatic hemorrhage and chain of survival. Scand J Trauma Resusc Emerg Med. BioMed Central Ltd; 2023. 10.1186/s13049-023-01088-8 PMC10207757 37226264

[4] GBD 2021 Causes of Death Collaborators. Global burden of 288 causes of death and life expectancy decomposition in 204 countries and territories and 811 subnational locations, 1990–2021: a systematic analysis for the global burden of disease study 2021. Lancet. 2024;403:2100–32. 38582094 10.1016/S0140-6736(24)00367-2 PMC11126520

[5] Heckbert SR, Vedder NB, Hoffman W, Winn RK, Hudson LD, Jurkovich GJ, et al. Outcome after hemorrhagic shock in trauma patients. J Trauma. 1998;45:545–9. 9751548 10.1097/00005373-199809000-00022

[6] Cannon JW, Khan MA, Raja AS, Cohen MJ, Como JJ, Cotton BA, et al. Damage control resuscitation in patients with severe traumatic hemorrhage: A practice management guideline from the Eastern association for the surgery of trauma. Journal of trauma.and acute care surgery. Lippincott Williams and Wilkins; 2017. pp. 605–17. 10.1097/TA.0000000000001333 28225743

[7] Wray JP, Bridwell RE, Schauer SG, Shackelford SA, Bebarta VS, Wright FL, et al. The diamond of death: hypocalcemia in trauma and resuscitation. American journal of emergency medicine. W.B. Saunders; 2021. pp. 104–9. 10.1016/j.ajem.2020.12.065 33421674

[8] Cherry RA, Bradburn E, Carney DE, Shaffer ML, Gabbay RA, Cooney RN. Do early ionized calcium levels really matter in trauma patients? J Trauma - Injury Infect Crit Care. 2006;61:774–9. 10.1097/01.ta.0000239516.49799.63 17033540

[9] Vivien B, Langeron O, Morell E, Devilliers C, Carli PA, Coriat P, et al. Early hypocalcemia in severe trauma. Crit Care Med. 2005;33:1946–52. 16148464 10.1097/01.ccm.0000171840.01892.36

[10] Magnotti LJ, Bradburn EH, Webb DL, Berry SD, Fischer PE, Zarzaur BL, et al. Admission ionized calcium levels predict the need for multiple transfusions: A prospective study of 591 critically ill trauma patient. J Trauma - Injury Infect Crit Care. 2011;70:391–7. 10.1097/TA.0b013e31820b5d98 21307739

[11] Vasudeva M, Mathew JK, Fitzgerald MC, Cheung Z, Mitra B. Hypocalcaemia and traumatic coagulopathy: an observational analysis. Vox Sang. 2020;115:189–95. 31845341 10.1111/vox.12875

[12] Webster S, Todd S, Redhead J, Wright C. Ionised calcium levels in major trauma patients who received blood in the emergency department. Emerg Med J. 2016;33:569–72. 26848163 10.1136/emermed-2015-205096

[13] Vasudeva M, Mathew JK, Groombridge C, Tee JW, Johnny CS, Maini A, et al. Hypocalcemia in trauma patients: A systematic review. J Trauma Acute Care Surg. 2021;90:396–402. 33196630 10.1097/TA.0000000000003027 PMC7850586

[14] Helsloot D, Fitzgerald M, Lefering R, Verelst S, Missant C. Trauma-induced disturbances in ionized calcium levels correlate parabolically with coagulopathy, transfusion, and mortality: a multicentre cohort analysis from the TraumaRegister DGU^®^. Crit Care. 2023;27. 10.1186/s13054-023-04541-3 PMC10324195 37415194

[15] Schaid TR, Lacroix I, Cohen MJ, Hansen KC, Moore EE, Sauaia A, et al. Metabolomic and proteomic changes in trauma-induced hypocalcemia. Shock. 2023;60:652–63. 37695733 10.1097/SHK.0000000000002220 PMC10841339

[16] Vettorello M, Altomare M, Spota A, Cioffi SPB, Rossmann M, Mingoli A et al. Early hypocalcemia in severe trauma: an independent risk factor for coagulopathy and massive transfusion. J Pers Med. 2023;13. 10.3390/jpm13010063 PMC9863326 36675724

[17] Liaud-Laval G, Libert N, Pissot M, Chrisment A, Ponsin P, Boutonnet M et al. Severe hypocalcemia at admission is associated with increased transfusion requirements: A retrospective study in a level 1 trauma center. Injury. 2024;55. 10.1016/j.injury.2023.111168 37926665

[18] Page MJ, McKenzie JE, Bossuyt PM, Boutron I, Hoffmann TC, Mulrow CD, et al. The PRISMA 2020 statement: an updated guideline for reporting systematic reviews. PLoS Med. 2021;18:e1003583. 33780438 10.1371/journal.pmed.1003583 PMC8007028

[19] Ouzzani M, Hammady H, Fedorowicz Z, Elmagarmid A. Rayyan-a web and mobile app for systematic reviews. Syst Rev. 2016;5:210. 27919275 10.1186/s13643-016-0384-4 PMC5139140

[20] Munn Z, Moola S, Lisy K, Riitano D, Tufanaru C. Methodological guidance for systematic reviews of observational epidemiological studies reporting prevalence and cumulative incidence data. Int J Evid Based Healthc. 2015;13:147–53. 26317388 10.1097/XEB.0000000000000054

[21] Munn Z, Moola S, Riitano D, Lisy K. The development of a critical appraisal tool for use in systematic reviews addressing questions of prevalence. Int J Health Policy Manag. 2014;3:123–8. 25197676 10.15171/ijhpm.2014.71 PMC4154549

[22] Mahanna Ahmed JM, Ahmad Elewa GM, Mahmoud Zaki MS, Anis Said SG, Khalifa Ragab AAEG. Ionized hypocalcemia as a prognostic factor of early mortality in traumatic brain injury. Egypt J Anaesth. 2024;40:273–81.

[23] Young CC, Seong YH. The value of initial ionized calcium as a predictor of mortality and triage tool in adult trauma patients. J Korean Med Sci. 2008;23:700–5. 18756060 10.3346/jkms.2008.23.4.700 PMC2526411

[24] Byerly S, Inaba K, Biswas S, Wang E, Wong MD, Shulman I et al. Transfusion-Related Hypocalcemia After Trauma. World J Surg [Internet]. 2020;44:3743–50. Available from: https://onlinelibrary.wiley.com/doi/10.1007/s00268-020-05712-x 10.1007/s00268-020-05712-x PMC7391918 32734451

[25] Badarni K, Harush N, Andrawus E, Bahouth H, Bar-Lavie Y, Raz A, et al. Association between admission ionized calcium level and neurological outcome of patients with isolated severe traumatic brain injury: A retrospective cohort study. Neurocrit Care. 2023;39:386–98. 36854866 10.1007/s12028-023-01687-4

[26] Mekkodathil A, El-Menyar A, Hakim S, Al Jogol H, Parchani A, Peralta R et al. Initial serum levels of magnesium and calcium as predictors of mortality in traumatic brain injury patients: A retrospective study. Diagnostics. 2023;13. 10.3390/diagnostics13061172 PMC10047507 36980480

[27] Ciaraglia A, Lumbard D, DeLeon M, Barry L, Braverman M, Schauer S et al. Retrospective analysis of the effects of hypocalcemia in severely injured trauma patients. Injury. 2024;55. 10.1016/j.injury.2024.111386 38310003

[28] Rushton TJ, Tian DH, Baron A, Hess JR, Burns B. Hypocalcaemia upon arrival (HUA) in trauma patients who did and did not receive prehospital blood products: a systematic review and meta-analysis. Eur J Trauma Emerg Surg. 2024;50(6):3353. 10.1007/s00068-024-02454-6 PMC11458635 38319350

[29] Zivin JR, Gooley T, Zager RA, Ryan MJ. Hypocalcemia: a pervasive metabolic abnormality in the critically ill. Am J Kidney Dis. 2001;37:689–98. 11273867 10.1016/s0272-6386(01)80116-5

[30] Strecker W, Gebhard F, Perl M, Rager J, Buttenschön K, Kinzl L, et al. Biochemical characterization of individual injury pattern and injury severity. Injury. 2003;34:879–87. 14636727 10.1016/s0020-1383(03)00022-6

[31] Erkus S, Turgut A, Kalenderer O. Alterations in serum IL-6 levels in traumatized pediatric patients: A preliminary study for second hit concept. J Orthop Sci. 2022;27:440–7. 33549402 10.1016/j.jos.2020.12.023

[32] Koch SM, Mehlhorn U, Baggstrom E, Donovan D, Allen SJ. Hypercalcitoninemia and inappropriate calciuria in the acute trauma patient. J Crit Care. 1996;11:117–21. 8891962 10.1016/s0883-9441(96)90007-6

[33] Carlstedt F, Lind L, Joachimsson PO, Rastad J, Wide L, Ljunghall S. Circulating ionized calcium and parathyroid hormone levels following coronary artery by-pass surgery. Scand J Clin Lab Invest. 1999;59:47–53. 10206097 10.1080/00365519950185995

[34] MacKay EJ, Stubna MD, Holena DN, Reilly PM, Seamon MJ, Smith BP, et al. Abnormal calcium levels during trauma resuscitation are associated with increased mortality, increased blood product use, and greater hospital resource consumption: A pilot investigation. Anesth Analg. 2017;125:895–901. 28704250 10.1213/ANE.0000000000002312 PMC5918410

[35] Wongtanasarasin W, Siri-Angkul N, Wittayachamnankul B, Chattipakorn SC, Chattipakorn N. Mitochondrial dysfunction in fatal ventricular arrhythmias. Acta Physiologica. Blackwell Publishing Ltd; 2021. 10.1111/apha.13624 33555138

[36] Eisner DA, Caldwell JL, Kistamás K, Trafford AW. Calcium and Excitation-Contraction coupling in the heart. Circ Res. 2017;121:181–95. 28684623 10.1161/CIRCRESAHA.117.310230 PMC5497788

[37] Szent-Györgyi AG. Calcium regulation of muscle contraction. Biophys J. 1975;15:707–23. 806311 10.1016/S0006-3495(75)85849-8 PMC1334730

[38] Stenman A. Hypocalcemia and its Impact on Cardiovascular Health. Reports in Thyroid Research [Internet]. 2023 [cited 2024 Dec 10];7:1–2. Available from: https://www.hilarispublisher.com/open-access/hypocalcemia-and-its-impact-on-cardiovascular-health-102614.html

[39] Desai TK, Carlson RW, Thill-Baharozian M, Geheb MA. A direct relationship between ionized calcium and arterial pressure among patients in an intensive care unit. Crit Care Med. 1988;16:578–82. 3371020 10.1097/00003246-198806000-00002

[40] Kronstedt S, Roberts N, Ditzel R, Elder J, Steen A, Thompson K et al. Hypocalcemia as a predictor of mortality and transfusion. A scoping review of hypocalcemia in trauma and hemostatic resuscitation. Transfusion (Paris) [Internet]. 2022 [cited 2024 Dec 10];62:S158. Available from: https://pmc.ncbi.nlm.nih.gov/articles/PMC9545337/10.1111/trf.16965 PMC9545337 35748676

[41] Schauer S, Nicholson S, Wright F, Rizzo J, Long B, Aden J et al. 1568: CLINICAL ASSESSMENT OF LOW CALCIUM IN TRAUMA (CALCIUM): An interim analysis. Crit Care Med [Internet]. 2024 [cited 2024 Dec 10];52:S755–S755. Available from: https://journals.lww.com/ccmjournal/fulltext/2024/01001/1568__clinical_assessment_of_low_calcium_in_trauma.1511.aspx

[42] Schriner JB, Van Gent JM, Meledeo MA, Olson SD, Cotton BA, Cox CS, et al. Impact of transfused citrate on pathophysiology in massive transfusion. Crit Care Explor. 2023;5:e0925. 37275654 10.1097/CCE.0000000000000925 PMC10234463

