# Supplementary material for: Prevalence and outcomes of hypocalcemia on ED arrival in traumatic patients before blood transfusions: a systematic review and meta-analysis
Source: Scand J Trauma Resusc Emerg Med. 2025 Mar 17;33:43. doi: 10.1186/s13049-025-01361-y (PMC11916158; doi:10.1186/s13049-025-01361-y)

**Supplementary Files**

**Table S1.** Key inclusion and exclusion of included studies.

| **No** | **Study Country** | **Study type** | **Study duration** | **Setting** | **Key inclusion criteria** | **Key exclusion criteria** |
| --- | --- | --- | --- | --- | --- | --- |
| 1 | Vivien, 2005  France | Prospective observational cohort | 2002 | Level 1 academic trauma center | - Prehospital decision on severity  to level 1 trauma center | - Transferred from other hospital,  - Previous transfusion before arrival and/or before blood sampling,  - Prehospital administration of calcium or fluid loading contain calcium |
| 2 | Cherry, 2006  USA | Retrospective  observational cohort | 2000-2002 | Level 1 trauma center | - Trauma 1 trauma team activation  - hemodynamic instability,   - respiratory compromise,   - multiple patients arriving simultaneously,   - gunshot or stab wounds to the neck, chest, and/or abdomen,   - severe craniofacial fractures with extensive hemorrhage and/or airway compromise,  - acute paraplegia or quadriplegia,  - witnessed prehospital cardiopulmonary arrest with <15 minutes CPR for penetrating trauma and <5 minutes for blunt trauma | - Previous transfusion before arrival |
| 3 | Choi, 2008  Korea | Ambispective observational cohort | 2005 | University hospital | - altered mental status,  - hemodynamic instability,  - respiratory compromise  - severe craniofacial fractures with extensive hemorrhage and/or airway compromise,  - flail chest,  - any penetrating injuries to the head, neck, torso, or extremities proximal to the elbow and knee,  - limb paralysis,  - amputation proximal to wrist and ankle,  - two or more proximal long bone fractures  - pelvic bone fractures,  - falls of >6 meters,  - high speed auto crash, roll-over, or pedestrian run over. | - more than 24 hr of time interval from injury onset to arrival on EMC,  - known underlying liver cirrhosis,  - known underlying chronic renal failure,  - known parathyroid disease,  - current treatment for malignancy,  - age younger than 16 years. |
| 4 | Magnotti, 2011  USA | Prospective observational cohort | 2008 | Level 1 trauma center | (1) physiologic alterations including known GCS <14, core temperature < 28°C, or abnormal vital signs (systolic blood pressure < 90 mm Hg, pulse < 60 or >120 beats per minute, and respirations <10 or >30 breaths per minute),  (2) mechanism of injury (with physiologic stability) including strangulations/hangings, penetrating wounds to the neck, chest, and/or abdomen, penetrating wounds to the extremities with neurovascular deficit, fatality within vehicle, rollovers/ ejections from vehicle, pregnant or intoxicated patients when history is suggestive of major trauma, blunt trauma with complaints relative to the abdomen or thorax, extrication times >20 minutes and falls >15 ft;  (3) anatomic alterations including severe craniofacial fractures with extensive hemorrhage and/or airway compromise, spinal cord injuries, pelvic instability, significant hemorrhage, major crush injury, flail chest, open fractures, multiple long bone fractures, and major amputations | - Previous transfusion before arrival |
| 5 | Webster, 2016  UK | Retrospective  observational cohort | 2013-2014 | Trauma audit research network | - All major trauma patients received blood product including both adult and pediatric | - no documented blood product administration time,  - no documented ionized calcium level or no ionized calcium level before receiving blood in the ED |
| 6 | Vasudeva, 2019  Australia | Retrospective observational  cohort | 2014-2018 | Level 1 trauma center | - injury Severity Score (ISS) of >12,  - death after injury,  - admission to ICU for more than 24 h with mechanical ventilation,  - partial- and full-thickness burns ≥20% total body surface area,  - urgent surgery within 48 h from arrival such as craniotomy, thoracotomy, laparotomy, pelvic or spine surgery, or interventional radiology | - previous transfusion before hospital presentation  - age < 18 years of age  - shock index < 1 |
| 7 | Byerly, 2020  USA | Retrospective  observational cohort | 2004 | Level 1 trauma center | - all trauma patients presenting to the emergency department (ED) that had an ionized calcium level drawn within 48 h of arrival | - age < 18 years old |
| 8 | Helshoot, 2023  Multi-center | Retrospective observational cohort | 2015-2019 | Trauma registry DGU | - available data registered on DGU trauma registry,  - adult traumatic patients with abbreviated injury scale (AIS) ≥ 3,  - direct on admission from the accident scene to a European trauma | - age < 16 years old,  - transferred out to another hospital within 48 h,  - Patients with ‘basic dataset’ registration,  - iCa2+ levels were missing or considered invalid |
| 9 | Scahid Jr, 2023  USA | Prospective observational cohort | N/A | Urban level 1 trauma center | - Adults (aged ≥18 years) with highest-level trauma activation criteria at an urban level 1 trauma center,  - ionized Ca2+ measured on ED arrival | - previous transfusion before blood samples obtained,  - penetrating injuries (for subgroup analysis),  - isolated traumatic brain injury (for subgroup analysis) |
| 10 | Badarni, 2023  Israel | Retrospective observational cohort | 2014-2020 | Level 1 trauma center | - patients 16 years or older,  - isolated severe TBI; head injury with an abbreviated injury score (AIS ≥ 4),  - absence of severe (AIS > 2) extracranial injuries | - transferred from or to other hospitals,  - patients who received blood products before the first blood gas analysis,  - diagnosis of hyperparathyroidism or hypoparathyroidism,  - treatment with anticoagulants (LWMH, warfarin or novel oral anticoagulants) |
| 11 | Maekkodathil, 2023  Qatar | Retrospective observational cohort | 2016-2021 | Level 1 trauma center | - diagnosis of TBI according to ICD-10-CM in QTR databases (cases with fracture of the skull, fracture of other specified skull and facial bones, unspecified fracture, injury of the optic chiasm, injury of optic tract and pathways, injuries of visual cortex, intracranial injury, crushing injury of skull, and unspecified injury of the head) | - penetrating injuries,  - transferred from other hospitals,  - incomplete data |
| 12 | Vettorello, 2023  Italy | Retrospective observational cohort | 2015-2021 | Level 1 trauma center | - all trauma major patients presented directly from the scene  - injury severity score (ISS) ≥ 16 | - Patients aged < 18 years,  - transferred from another facility,  - who already received intravenous calcium  - all patients already transfused with pRBC  - out-of-hospital cardiocirculatory arrest  - missing ionized calcium data at the time of presentation or within 30 min from admission (before any infusion and blood product transfusion) |
| 13 | Ahmed, 2024  Egypt | Prospective observational cohort | 2022 | Level 1 trauma center | - adults (≥18 years old) who had been admitted to the hospitals with TBI | - refusal to sign the consent to participate in the study,  - physical state in ASA III and IV,  - TBI lasting more than a day,  - multisystem trauma, including exposed fractures, thoracic injuries, and abdominal organs like the liver or spleen that have been lacerated,  - conditions like hyperparathyroidism, acute pancreatitis, and hydrochlorothiazide therapy that impact calcium metabolism,  - pregnancy and other electrolyte disturbance as hyperphosphatemia and hypomagnesemia |
| 14 | Ciaraglia, 2024  USA | Retrospective observational cohort | 2016-2019 | Trauma registry | - adult patients (i.e., age >18 years) who sustained traumatic injury and were categorized as the institution’s highest level of trauma activation  - ionized calcium level drawn within 30 min of arrival to the emergency department | - isolated severe head injury,  - those who were pulseless on arrival or who died within 30 min of presentation,  - transfers from other hospitals,  - vulnerable populations |
| 15 | Liaud-Laval, 2024  France | Retrospective observational cohort | 2015-2021 | Level 1 trauma center | - all patients arrived directly from the scene of injury in an ambulance with an emergency physician on board  - site-specific trauma activation protocols in the trauma department  - patients died during initial management  - those who were admitted to the intensive care unit  - whose iCa on arrival was measured  - who received at least one BP during the first 24 h after hospital admission | - patients under 18 years of age,  - detained patients,  - pregnant women |

**Table S2.** Quality assessment using the Joanna Briggs Institute’s critical appraisal checklist of included studies.

| Joanna Briggs Institute’s critical appraisal checklist | Vivien, 2005 | Cherry, 2006 | Choi, 2008 | Magnotti, 2011 | Webster, 2016 | Vasudeva, 2019 | Byerly, 2020 | Helshoot, 2023 | Scahid Jr, 2023 | Badarni, 2023 | Maekkodathil, 2023 | Vettorello, 2023 | Ahmed, 2024 | Ciaraglia, 2024 | Liaud-Laval, 2024 |
| --- | --- | --- | --- | --- | --- | --- | --- | --- | --- | --- | --- | --- | --- | --- | --- |
| Was the sample frame appropriate to address the target population? | Yes | Yes | Yes | Yes | Unclear | Yes | Yes | Yes | Yes | Unclear | Yes | Yes | Unclear | Yes | Yes |
| Were study participants sampled in an appropriate way? | Yes | Yes | Yes | Yes | Yes | Yes | Yes | Yes | Yes | Unclear | Unclear | Yes | Yes | Yes | Yes |
| Was the sample size adequate? | Unclear | Yes | Yes | Yes | No | Yes | Yes | Yes | No | Unclear | Yes | Yes | No | Yes | Yes |
| Were the study subjects and the setting described in detail? | Yes | Unclear | Yes | Yes | Yes | Yes | Yes | Yes | Yes | Yes | Yes | Yes | Yes | Yes | Yes |
| Was the data analysis conducted with sufficient coverage of the identified sample? | Unclear | Unclear | Unclear | Yes | Unclear | Yes | Yes | Unclear | Unclear | Unclear | Yes | Unclear | Unclear | Yes | Yes |
| Were valid methods used for the identification of the condition? | Yes | Yes | Yes | Yes | Yes | Yes | Yes | Yes | Yes | Yes | Unclear | Yes | Yes | Yes | Yes |
| Was the condition measured in a standard, reliable way for all participants? | Yes | Yes | Yes | Yes | Yes | Yes | Yes | Yes | Yes | Yes | Yes | Yes | Yes | Yes | Yes |
| Was there appropriate statistical analysis? | Yes | Yes | Yes | Yes | Unclear | Yes | Yes | Yes | Yes | Yes | Yes | Yes | Unclear | Yes | Yes |
| Was the response rate adequate, and if not, was the low response rate managed properly? | Yes | Yes | Yes | Yes | Yes | Yes | Yes | Yes | Yes | Yes | Yes | Yes | Yes | Yes | Yes |
| Total score (out of 9) | **7** | **7** | **8** | **9** | **5** | **9** | **9** | **8** | **7** | **5** | **7** | **8** | **5** | **9** | **9** |

**Table S3.** Search strategies for each database.

| Sources | Search terms | Results |
| --- | --- | --- |
| PubMed | ("Hypocalcemia"[Mesh]) AND ("Wounds and Injuries"[Mesh] OR trauma) AND (("prevalence") OR ("incidence") OR ("Epidemiology"[Mesh])) | 93 |
| Embase | injury/exp AND (hypocalcemia/exp OR 'hypocalcaemia' OR 'hypocalcaemic activity' OR 'hypocalcemia' OR 'hypocalcemic activity' OR 'hypocalcinemia' OR 'secondary hypocalcaemia' OR 'secondary hypocalcemia') AND (incidence/exp OR prevalence/exp) | 547 |
| Web of Science | ((ALL=(trauma) OR ALL=(injury)) AND ALL=(hypocalcemia)) NOT DT=(Abstract of Published Item OR Book Chapter OR Book Review OR Correction OR Meeting Abstract OR Review) | 771 |
| Scopus | trauma* AND ( hypocalcemia OR hypocalcemic ) | 573 |

**Figure S1.** Forest plot of incidence of severe hypocalcemia in traumatic adult patients, which subgroups as some concern and low risk of bias.


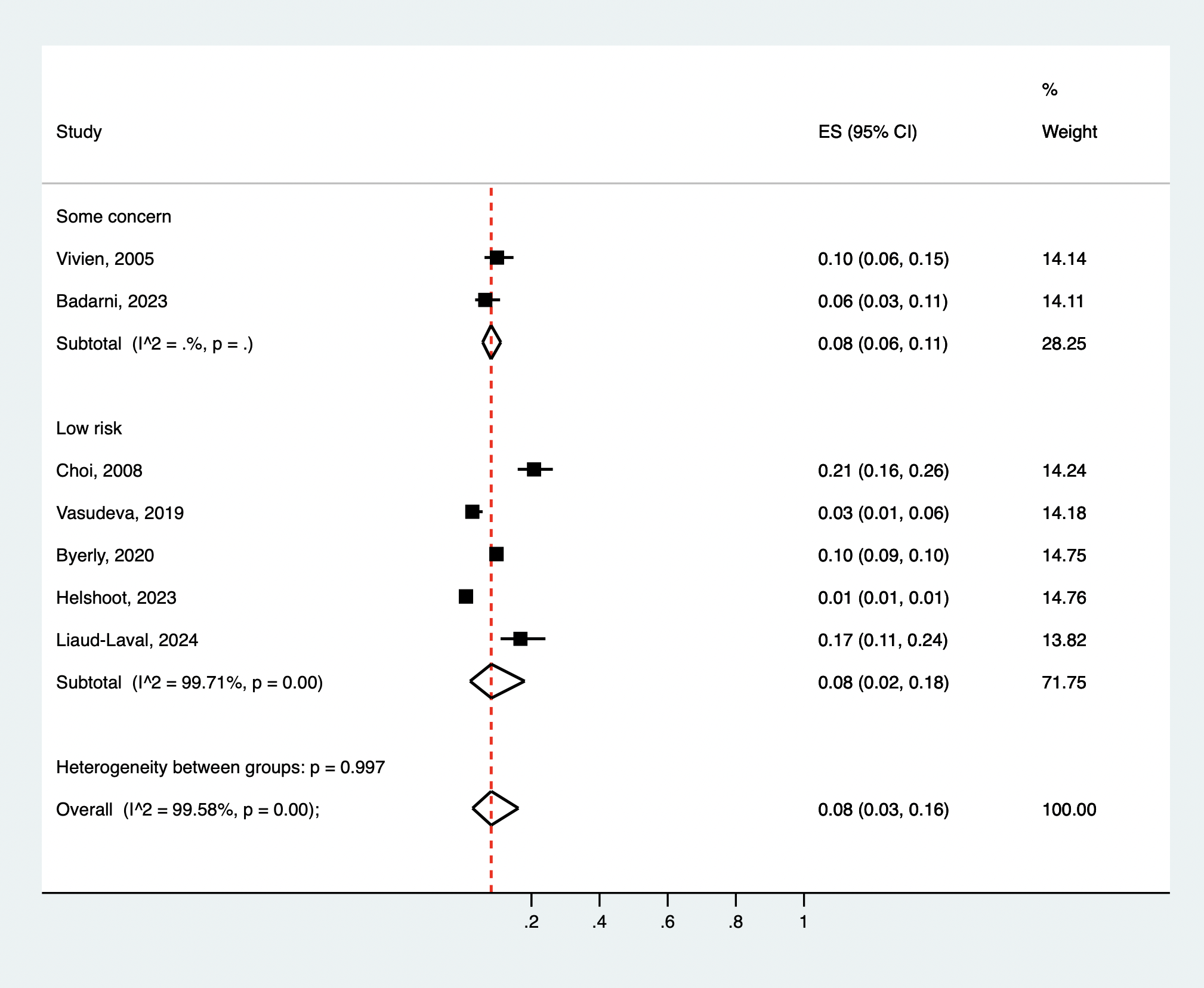


**Figure S2.** Forest plot of severe hypocalcemia and mortality rate in traumatic adult patients using a random-effects model, which subgroups as some concern and low risk of bias.


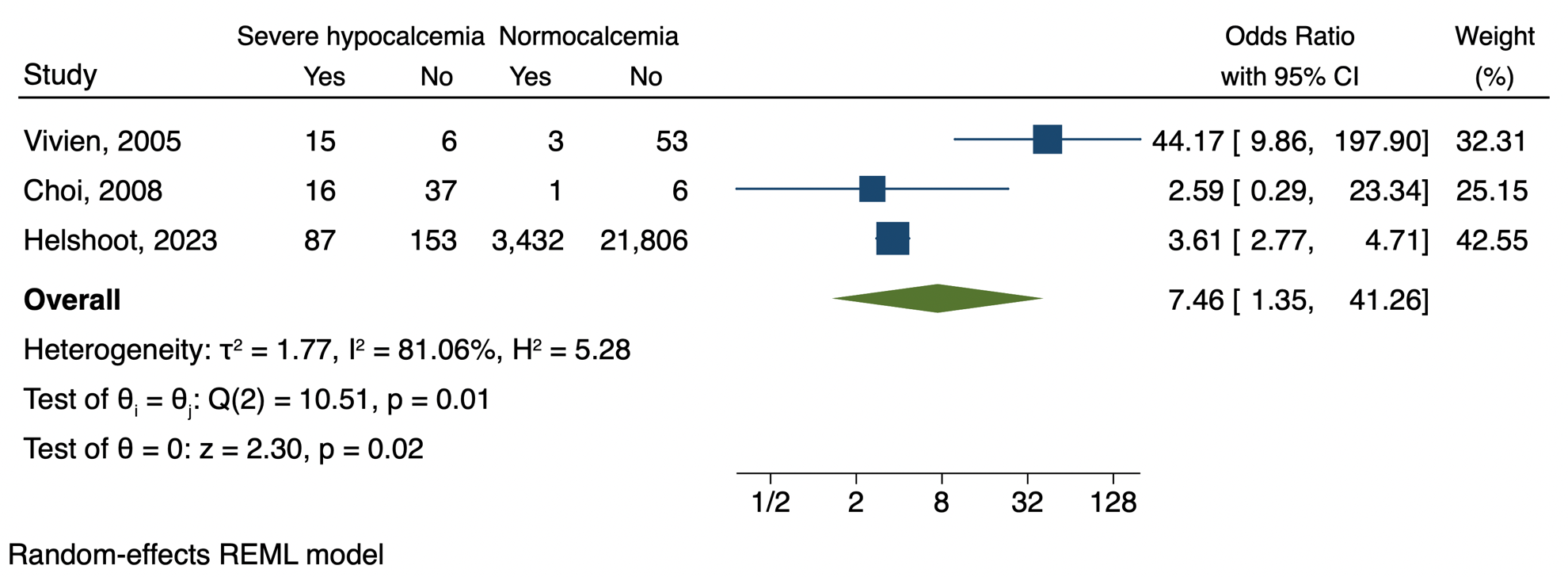

Supplement: Supplementary file 1 — Supplementary Material 1 [file 13049_2025_1361_MOESM1_ESM.docx]
